# Primary teeth microhardness and lead (Pb) levels

**DOI:** 10.1016/j.heliyon.2019.e01551

**Published:** 2019-04-24

**Authors:** Betsy Foxman, Ethan Kolderman, Elizabeth Salzman, Anna Cronenwett, Carlos Gonzalez-Cabezas, Katherine Neiswanger, Mary L. Marazita

**Affiliations:** a Center for Molecular and Clinical Epidemiology of Infectious Diseases, Department of Epidemiology, University of Michigan School of Public Health, 1415 Washington Heights, Ann Arbor, Michigan, 48109, USA; b University of Michigan School of Dentistry, 101 N. University Ave, Ann Arbor, Michigan, 48109, USA; c Department of Cariology, Restorative Sciences and Endodontics, School of Dentistry, University of Michigan School of Dentistry, 101 N. University Ave, Ann Arbor, Michigan, 48109, USA; d Center for Craniofacial and Dental Genetics, Department of Oral Biology, School of Dental Medicine, University of Pittsburgh, 3501 Terrace Street, Pittsburgh, Pennsylvania, 15261, USA; e Department of Human Genetics, Graduate School of Public Health, Clinical and Translational Sciences Institute, School of Medicine University of Pittsburgh, 130 De Soto Street, Pittsburgh, Pennsylvania, 15261, USA

**Keywords:** Dentistry

## Abstract

**Objectives:**

Lead (Pb) exposure is associated with dental caries. Whether Pb affects tooth microhardness, is unclear. Our objective was to assess whether Pb concentration is associated with microhardness.

**Methods:**

Exfoliated primary teeth were collected from 46 volunteers. Teeth were sectioned, one half of each tooth was tested for enamel Knoop microhardness. The remaining half was digested and Pb measured using an inductively coupled plasma-mass spectrometer.

**Results:**

The correlations between Pb levels and microhardness were very low, and were not statistically significant at p < 0.05.

**Conclusions:**

Previous exposure to high levels of Pb was not associated with decreased tooth microhardness.

**Clinical significance:**

This study assessed whether Pb in deciduous teeth is associated with tooth microhardness. As this was not the case, further studies are needed to identify the mechanisms behind the association between lead exposure and tooth decay.

## 1 Introduction

Childhood caries are a significant public health problem in the United States [1], recognized as a top priority of the American Academy of Pediatrics [2]. Among US children aged 6–8 years, there are significant racial/ethnic and social disparities in the prevalence of decay in primary teeth: prevalences among Hispanics and African Americans (19.4% and 20.5%, respectively) are roughly double the prevalence among whites (10.1%) [3]; further, those whose parents have less education or lower incomes are more likely to have untreated tooth decay and loss [4, 5]. Diet and dental hygiene in childhood, however, only partially explain disparities in early onset of childhood caries [6].

There have been several studies suggesting that environmental exposure to lead (Pb) also contributes to disparities in childhood caries, particularly in primary teeth [7, 8, 9, 10, 11, 12, 13]. A 2017 study of 1,564 Korean children showed that each μg/dL of blood Pb was associated with a 1.16 (95% CI: 0.91–1.49) greater prevalence of decayed, missing and filled surfaces in deciduous, but not permanent teeth, after adjustment for age, sex, mother's education, household income and urinary cotinine [7]. Further, in an Egyptian study of teeth from children and adults, Pb levels were higher in the teeth pulp from carious teeth (n = 62) than healthy teeth (n = 39) (77 versus 29 ppm, p = 0.004). An analysis of children participating in the Third National Health and Nutrition Examination Survey (NHANES) estimated that among 5–17-year olds, 13.5% of dental caries occurring among children exposed to high lead levels and 9.6% of dental caries among children exposed to moderate lead levels, can be attributed to lead exposure [11].

One hypothesized mechanism for the association of Pb with dental caries focuses on the impact of Pb on dental enamel formation. Children exposed to Pb have higher salivary Pb levels [14]. Salivary Pb is incorporated in hydroxyapatite crystals substituting for calcium ions, leading to enamel hypoplasia, increased abrasion, and discoloration [15]. In an animal model, Knoop microhardness levels were inversely associated with lead exposure in regions of maturing dental enamel but not fully mature enamel [16]. Consistent with this finding, Ghadimi *et al.* did not find an association of Pb with microhardness, but showed that Pb concentration was associated with size of apatite nanocrystals in sound, extracted upper anterior teeth; whether the teeth were primary or permanent was not reported [17]. The current study directly addresses whether Pb concentration is associated with microhardness using 46 exfoliated deciduous (primary) teeth.

## 2 Materials and methods

### 2.1 Tooth collection

Exfoliated primary teeth were collected from volunteers. Participants were instructed to note the approximate date of tooth loss, and to mail the teeth in pre-addressed, stamped envelopes to the Center for Craniofacial and Dental Genetics (CCDG) at the University of Pittsburgh. No personal identifying information was requested. Teeth were maintained dry by participants and researchers. Teeth were catalogued and examined for presence of tooth decay by a licensed dental hygienist. The study protocol was deemed exempt by the University of Pittsburgh Institutional Review Board.

### 2.2 Microhardness

All teeth were longitudinally sectioned in the buccal-lingual direction using a South Bay Technology, Inc. Model 650 Low Speed Diamond Wheel Saw™ at the University of Michigan Dental School. Teeth were sectioned in wax using a blade continuously running through deionized water. One section of each tooth was used for lead detection as described, below. Sections used for microhardness testing were progressively polished using 240 grit, 400 grit, 600 grit, and 1200 grit sandpaper for 90 seconds respectively. Enamel microhardness was measured using a Knoop microhardness tester. Measurements were made starting 20 μm from the dental enamel junction (DEJ) moving in 20 μm increments towards the enamel surface. The load was 50 grams for 10 seconds for each test. Each tooth's microhardness was measured in 3 series of measurements containing 10 readings each (Initial measurement 20 μm coronal of DEJ; final measurement 200 μm coronal of DEJ). Each new series of measurements was started 100 μm lateral to previous series.

### 2.3 Lead detection

The remaining section of each tooth (i.e. not prepared for microhardness testing) was digested to detect Pb levels, using modifications of the method described by Amr and Helal (2010) to meet equipment specifications, by the Trace Metals Unit at the Michigan Department of Health and Human Services, Bureau of Laboratories. The concentrations of Pb were optimized using a standard ^193^lr solution at a concentration of 10 ppb in 2% HNO_3_. Following each measurement, tubes were cleaned using 2% HNO_3_. An ESI MicroFlow PFA-ST3-84 nebulizer was used for the inductively coupled plasma-mass spectrometer measurements. Teeth were weighed, and then digested in 5 mL of HNO_3_ using a CEM Discover SPD microwave digestion system. Three quality controls using certified material (low: 963.11, %CV 5.31; medium: 7622.08, %CV 5.18; and high: 13374.64, %CV 3.98, in ppb) were included at the beginning of each run right after the calibration curve before participant specimens were assessed.

### 2.4 Statistical analysis

We estimated the Pearson correlation between parts per billion of Pb and microhardness at 20, 40, 60, 200 micrometers and the overall average (measured in Knoop). To test whether mean Pb levels and tooth hardness varied by time we used a two sample t test.

## 3 Results

**Fig. 1:**
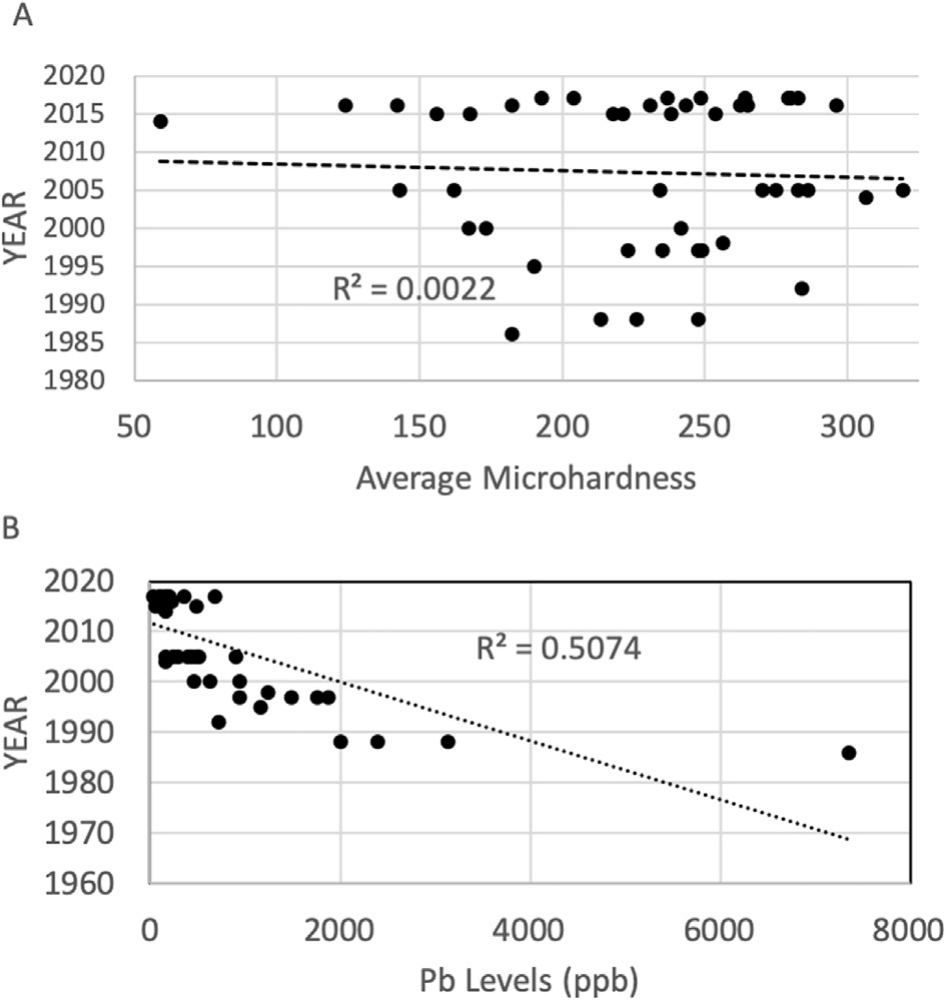
A) Average microhardness (Knoop) and B) average Pb levels (parts per billion) by year tooth was exfoliated (n = 46 sound primary teeth). Note: R^2^ for Pb by year tooth was exfoliated after removing outlier is 0.69.

We collected 14 sound molars, 28 sound incisors, and 4 sound canines. Teeth were exfoliated between 1986 and 2017. The average Pb level was 736.7 ppbb, STD 1214.7, range 38.8–7355.5. There was no association between year exfoliated and microhardness (r = −0.047; p = 0.38) (Fig. 1a), but teeth exfoliated more recently had significantly lower Pb levels (r = −0.71, p < 0.001), particularly those exfoliated after 1999 (mean 280.4 versus 2188.4 ppb) (Fig. 1b). Pb was banned from gasoline in the United States in 1990 so this is not entirely unexpected. However, since we could not rule out that storage might have been associated with lead exposure we analyzed the data including and removing teeth exfoliated prior to 2000, and the results were the same (data not shown).

**Fig. 2:**
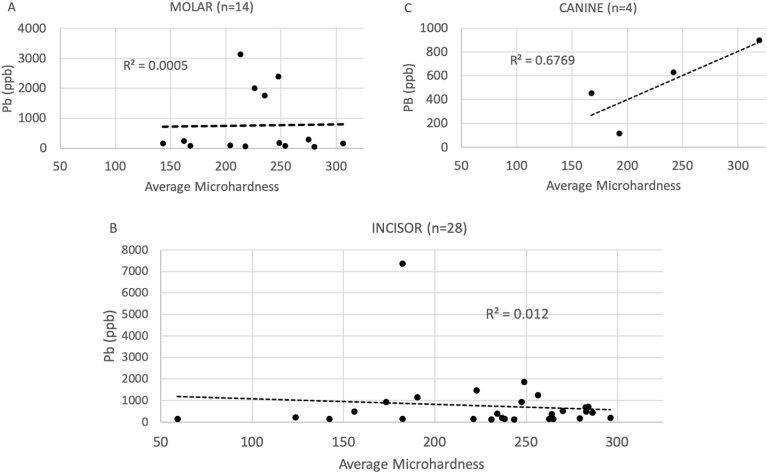
Average microhardness (Knoop) by Pb levels in parts per billion (ppb) and tooth type: A) Molar; B) Incisor; C) Canine. Forty-six sound exfoliated primary teeth.

The association between lead levels and microhardness varied little by where microhardness was measured (closest to the dental enamel junction or the tooth surface). Therefore, we present only the results using average microhardness. However, the associations between lead levels and microhardness did vary slightly by tooth type. Fig. 2 shows the average microhardness for all microhardness measures by Pb levels for each tooth type. For molars and incisors, the correlations were very low (R^2^ = 0.0005 and R^2^ = 0.012, respectively) and neither were statistically significant at p < 0.05. There was a strong positive correlation between lead levels and microhardness for canine teeth at 200 mm from the dental enamel junction (R^2^ = 0.68), but the sample size was small (n = 4), so although the result approached statistical significance (p = 0.06) we cannot rule out an alpha error, particularly as the correlations between lead levels and microhardness for molars and incisors were low, inconsistent, and were not statistically significant despite a larger sample size. A reanalysis excluding teeth exfoliated prior to 2000 did not change the results.

## 4 Discussion & conclusion

In contrast to results from an animal model which suggested that Pb exposure during enamel formation results in decreased microhardness thus potentially increasing risk of dental decay, we found no association between Pb levels and tooth microhardness in 46 exfoliated human primary teeth. The Pb levels observed here are lower, but overlap in range to those reported in a 2010 Egyptian study of 64 healthy primary teeth, where the average levels were 1200 ± 840 ppb (range 340–4,010) [18]. This may be due to the relatively small sample size included in each study and differences in exposure between the United States and Egypt.

As both fluoride and Pb are incorporated in hydroxyapatite crystals (Pb substituting for calcium ions, fluoride for hydroxyls), it is possible that when both are present there may be interactions leading to increase or decrease in tooth decay depending on the relative concentrations. A limitation of our study is that we have no measure of fluoride levels. At least one animal study suggests there might be an interaction between fluoride and Pb resulting in changes in enamel defects. Wistar rats were assigned to water with 1) 0.1 ppm fluoride, 2) 100 ppm fluoride, 3) 30 ppm Pb, or 4) 100 ppm fluoride and 30 ppm Pb. Rats exposed to 100 ppm fluoride and 30 ppm Pb had significantly more severe enamel defects than those exposed to 100 ppm fluoride alone (p < 0.0001) [19]. However, in a study of desalivated Sprague-Dawley rats exposed to 15 ppm fluoride and 10 or 25 ppm Pb, the protective effects of fluoride on dental decay did not change with Pb exposure [20]. In human studies, the association of Pb, fluoride exposure, and dental caries is largely unexplored. Thus, it remains an open question whether fluoride use mitigates the effects of Pb on dental caries. A second limitation of our study is we only measured overall Pb levels and cannot comment on the effects of Pb in different tooth structures on tooth hardness.

In conclusion, results from the current study do not suggest that Pb contamination leads to decreased tooth microhardness.

## Declarations

### Author contribution statement

Betsy Foxman: Conceived and designed the experiments; Analyzed and interpreted the data; Wrote the paper.

Ethan Kolderman: Performed the experiments; Analyzed and interpreted the data.

Elizabeth Salzman, Anna Cronenwett, Katherine Neiswanger, Mary L. Marazita: Analyzed and interpreted the data.

Carlos Gonzalez-Cabezas: Analyzed and interpreted the data; Contributed reagents, materials, analysis tools or data.

### Funding statement

This work was supported by the National Institutes of Health, grant DE014899.

### Competing interest statement

The authors declare no conflict of interest.

### Additional information

No additional information is available for this paper.
